# Discovery of deep-water coral frameworks in the northern Red Sea waters of Saudi Arabia

**DOI:** 10.1038/s41598-020-72344-5

**Published:** 2020-09-18

**Authors:** Mohammad A. Qurban, Periyadan K. Krishnakumar, Thadickal V. Joydas, Karuppasamy P. Manikandan, T. T. M. Ashraf, Goutham Sampath, Duraisamy Thiyagarajan, Song He, Stephen D. Cairns

**Affiliations:** 1 Center for Environment & Water, Research Institute, King Fahd University of Petroleum and Minerals (KFUPM), P. B. No. 391, Dhahran, 31261 Kingdom of Saudi Arabia; 2 National Center for Wildlife, Ministry of Environment, Water and Agriculture, Riyadh, Kingdom of Saudi Arabia; 3 Red Sea Research Center, Division of Biological and Environmental Science and Engineering, King Abdullah University of Science and Technology, Thuwal, Kingdom of Saudi Arabia; 4 Department of Invertebrate Zoology, National Museum of Natural History, Smithsonian Institution, Washington, DC 20560 USA

**Keywords:** Ocean sciences, Marine biology

## Abstract

This paper reports a deep-water coral framework (a single colonial bush or a larger bioconstruction of coral covering the sea bottom), formed entirely by the scleractinian coral *Eguchipsammia fistula* (Alcock, 1902) (Dendrophylliidae), in the northern Red Sea waters of Saudi Arabia at a depth of about 640 m. The framework consists of mostly live corals with a total area of about 10 m^2^ and the length of the individual coral branches range from 12 to 30 cm. Although *E. fistula* is ubiquitous, this discovery is the second record of a framework formed by this species and the first discovery of a large living reef in the Red Sea. The results of the genetic study indicate the potential existence of a genetic variation of *E. fistula* in the Red Sea. This discovery implies that the Red Sea has favorable habitats for framework-forming DWC species and highlights the need for conducting more systematic surveys for understanding their distribution, abundance, and ecology.

## Introduction

Azooxanthellate deep-water corals (DWCs) are reported from the tropics to the polar seas, occurring on continental margins and topographical high points throughout much of the world’s oceans^1,2^. A majority (around 74%) of the DWCs is solitary in habit, while the remainder (around 26%) are colonial^3^. Despite its highly saline and warm deep waters, the Red Sea is known for its diverse benthic habitats. Shallow-water coral reefs span the entire coastline of the Red Sea and are known for their high productivity, biodiversity, and endemism^4^. The occurrence of azooxanthellate deep-water corals has been only seldom reported from the warm, saline, and oxygen- and food-deprived environment of the Red Sea^5–8^. Several frame-building corals, including azooxanthellate species, have been reported from the twilight zone (100–210 m) of the Gulf of Aqaba in the Red Sea^9,10^. Their range of distribution depth is in the transient zone for coral distribution, which includes the upper distribution limits of a few DWCs and the lower distribution limits of several shallower water species^11^.

**Figure 1:**
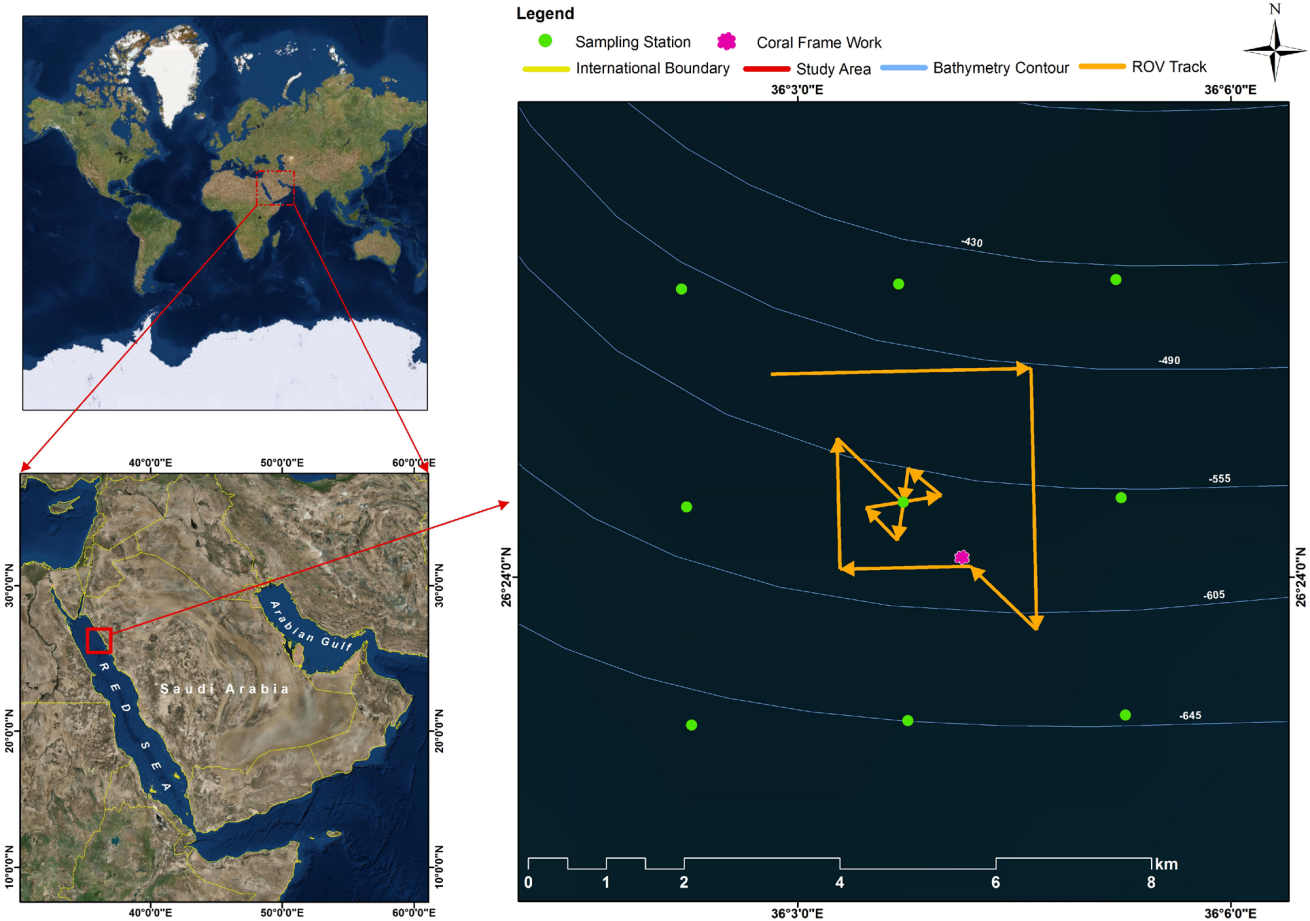
Location map of the Study Area (Basemap: Google) showing the tracks of ROV dives (orange lines) performed and the position of the observed coral framework (pink polygon), and stations (green points) used for water quality studies. Maps were created using ArcGIS 10.2 software by ESRI (www.arcgis.com). ArcGIS and ArcMap are the intellectual property of ESRI.

**Figure 2:**
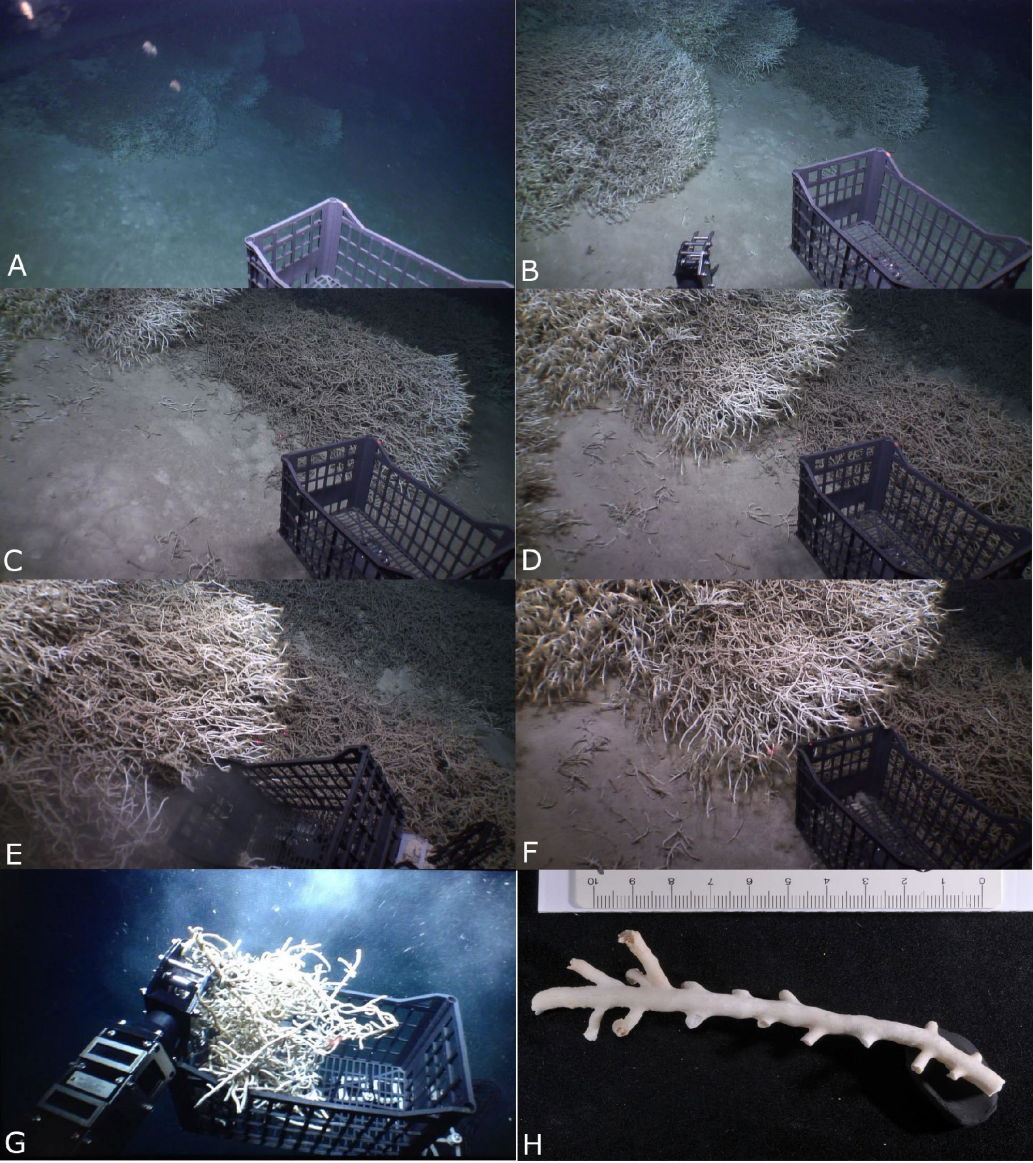
In situ photographs showing the habitat view of the *Eguchipsammia fistula* framework (distance between two red laser points is 10 cm). (**A**, **B**) Overall view of the coral framework recorded by the ROV. (**C**–**F)** In situ photographs of the framework taken from different angles. (**G**) Specimen collection using the ROV. (**H**) Corallum of *E. fistula* photographed in the laboratory.

**Figure 3:**
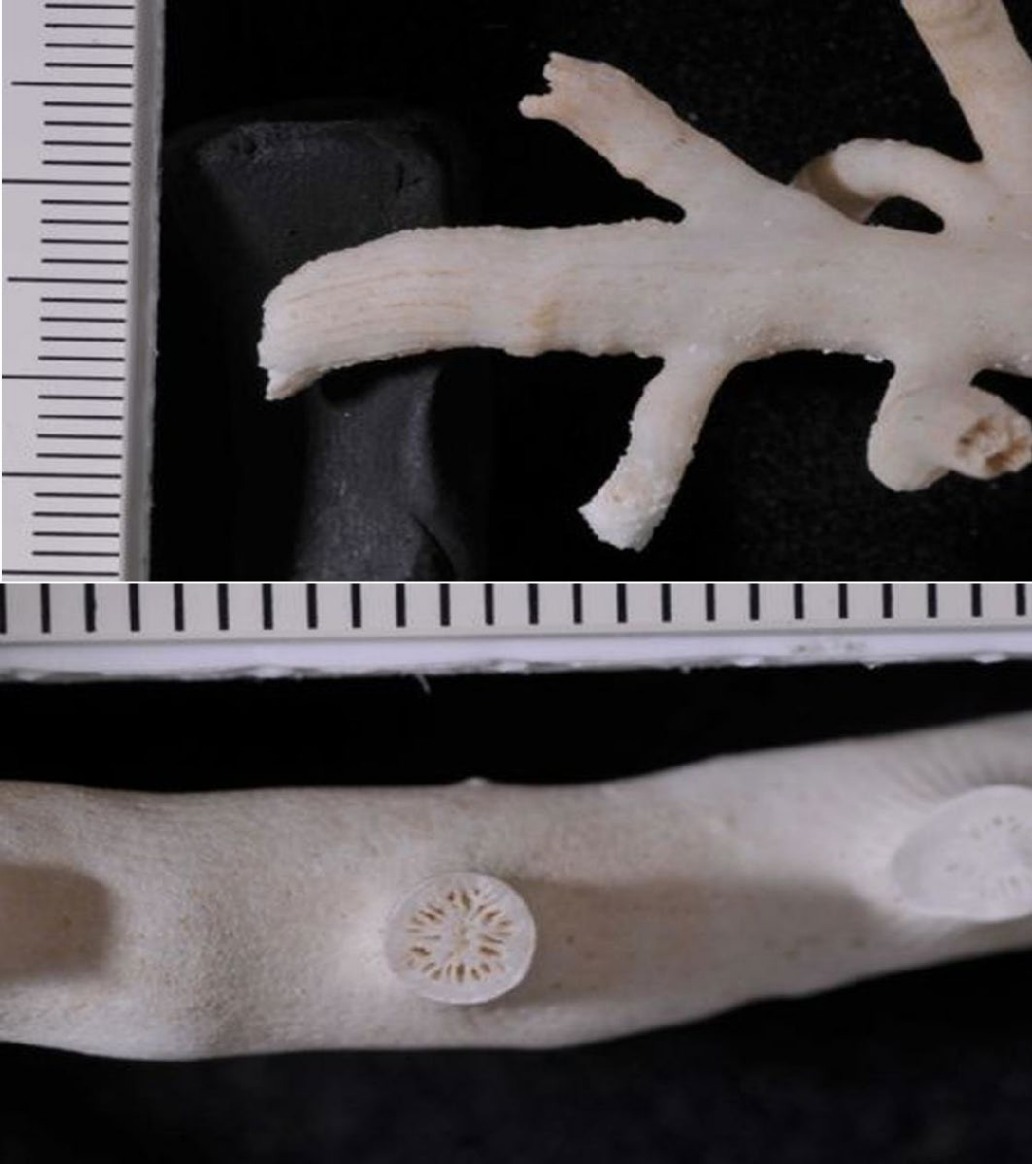
*Eguchipsammia fistula,* Dendrophylliidae*.* Specimen photographed in the laboratory showing the lateral view of a distal branch (top) and of an individual calice (bottom).

Here, a live DWC framework that was observed at a depth of about 640 m (Latitude: 26° 24.124′ N; Longitude: 36° 04.139′ E) and at a distance of ~ 26 km from the nearest shoreline in the northern Red Sea (Fig. 1) is reported for the first time. This live DWC framework was observed during an expedition in June 2014 using the Research Vessel R. V. *Aegaeo*, belonging to the Hellenic Centre for Marine Research. The framework is exclusively formed by the DWC species *Eguchipsammia fistula* (Alcock, 1902^12^) (Figs. 2 and 3), which was identified according to the morphological descriptions by Zibrowius^13^ and Cairns^14^, and further complemented by molecular data (See Supplementary information). The total area of the observed framework was around 10 m^2^ and the length of each coral branch ranged from 12 to 30 cm, mostly live with no or only a few instances of dead colonies along the periphery of the framework (Fig. 2). In July 2013, Tempera et al.^15^ reported a similar framework (reef) formed by *Eguchipsammia* (Dendrophylliidae) found off the Faial-Pico Channel (Azores, Northeast Atlantic) using the manned submersible *Lula 1,000*. The discovery reported here is the second record of such a living *Eguchipsammia* framework worldwide, suggesting that the Red Sea provides a habitat suitable for framework forming DWC species.

*E. fistula* is present throughout the Indo-West Pacific ^16–19^, and Marenzeller^7^ first discovered it in the Red Sea during the *Pola* expedition from 1895 to 1897. Recently, researchers have recorded scattered small colonies of this species again in the Saudi waters of the Red Sea^5,6^. Subsequent studies have revealed the ability of this coral species to cope with rapid and prolonged environmental changes, indicating a wide physiological plasticity, thereby providing reasons for its widespread distribution^20,21^. Climate change will alter the water temperature and chemistry of the oceans and negatively impact the shallow water and deep water coral reefs^22^. The combined effects of high temperatures and low oxygenation limit the proliferation of DWCs in the deep sea environment^23^. The Red Sea is in a stage of intense and abrupt warming, exhibiting an increase of 0.7 °C in the last decade^24^ with an increase of 3.45 °C projected over 2010–2099^25^. Cantin et al.^26^ have reported declining growth and calcification rates of coral colonies in the Red Sea. Considering the potential impacts of climate change, it is necessary to estimate the vulnerability of *E. fistula* to temperature changes. Seawater temperature affects the distribution of azooxanthellate DWCs, with most of the species living within the temperature range of ∼4–14°C^27,28^. Unlike most DWCs, these species in the Red Sea live at considerably higher temperatures (> 20 °C). Corals in the family Dendrophylliidae, (*Dendrophyllia cornigera, D. ramea*, *E*. *fistula* etc.) apparently have a natural tolerance to warm conditions^29^. The average seawater temperature measured in the vicinity of the *E. fistula* coral framework in this study is 21.5 °C (Table 1) and it is comparable to the temperature recorded at the Red Sea stations where this species were recorded earlier^5,6^. *E. fistula* coral specimens demonstrate a wide physiological plasticity and adapt well to basic aquaria systems when transferred from the deep water habitat in the Red Sea into a rearing system^20^. Deep-sea corals such as *E. fistula* in the Red Sea are acclimatized to the warm, saline, and low oxygen environment, and compared to their counterparts from other parts of the globe, they are better suited for adapting to climate change.

**Table 1:** Summary of the physio-chemical parameters recorded at stations (Fig. 1) where the deep-water coral framework was found.

Parameters	Surface (average ± SD)	Bottom (700 m) (average ± SD)
Current speed (cm s^−1^)	36.95 ± 8.21	8.70 ± 6.44
Current direction	WNW	WSW
Temperature (°C)	29.06 ± 0.17	21.47 ± 0.003
Salinity (PSU)	39.97 ± 0.03	40.54 ± 0.002
Density (kg m^−3^)	25.78 ± 0.06	28.59 ± 0.002
Dissolved Oxygen (mg L^−1^)	6.02 ± 0.06	1.83 ± 0.11
Nitrate (µmol L^−1^)	0.45 ± 0.88	5.95 ± 4.08
Nitrite (µmol L^−1^)	0.03 ± 0.007	0.07 ± 0.15
Phosphate (µmol L^−1^)	0.02 ± 0.001	0.50 ± 0.27
Silicon (µmol L^−1^)	0.51 ± 2.00	63.4 ± 0.68
Chlorophyll a (µg L^−1^)	0.17 ± 0.03	0.32 ± 0.12 (65 m depth)
Total Suspended Solids (mg L^−1^)	9.7 ± 1.65	10.0 ± 1.50

Taxonomic classification of the specimen was confirmed by the phylogenetic analyses of partial sequences of the mitochondrial 16S ribosomal RNA encoding gene. The 16S sequences determined in this study match (96.51% identical) the 16S sequence of *Eguchipsammia fistula* deposited in GenBank (JX629250), determined in corals collected from the deep Red Sea^5^. However, there is a 3.49% **(**100–96.51%**)** divergence in the 16S fragments between samples used in this study and that in *E. fistula* specimens previously sampled in the Red Sea^5^, indicating a new haplotype of this species and the existence of a genetic variation within the Red Sea populations. An IGR sequence from *Eguchipsammia fistula* is not available in the GenBank, and the IGR sequence obtained in this study is considered as the first record on this species. The coral *E. fistula* has a cosmopolitan distribution, and is known to occur in the Indo-Pacific, Australia, and New Zealand^30^. Most of the DWCs have been reported from the glacial Red Sea and they went extinct in response to unfavourable basin-wide hyperhaline conditions during the Last Glacial Maximum^8^. However, during the Holocene period, several bathyl taxa including the DWCs, have moved from the western Indian Ocean through the narrow and shallow sill of Bab al Mandab and successfully recolonized the Red Sea basin^8^. Hence, the *E. fistula* population in the Red Sea apparently represents a recent immigration from the western Indian Ocean.

**Figure 4:**
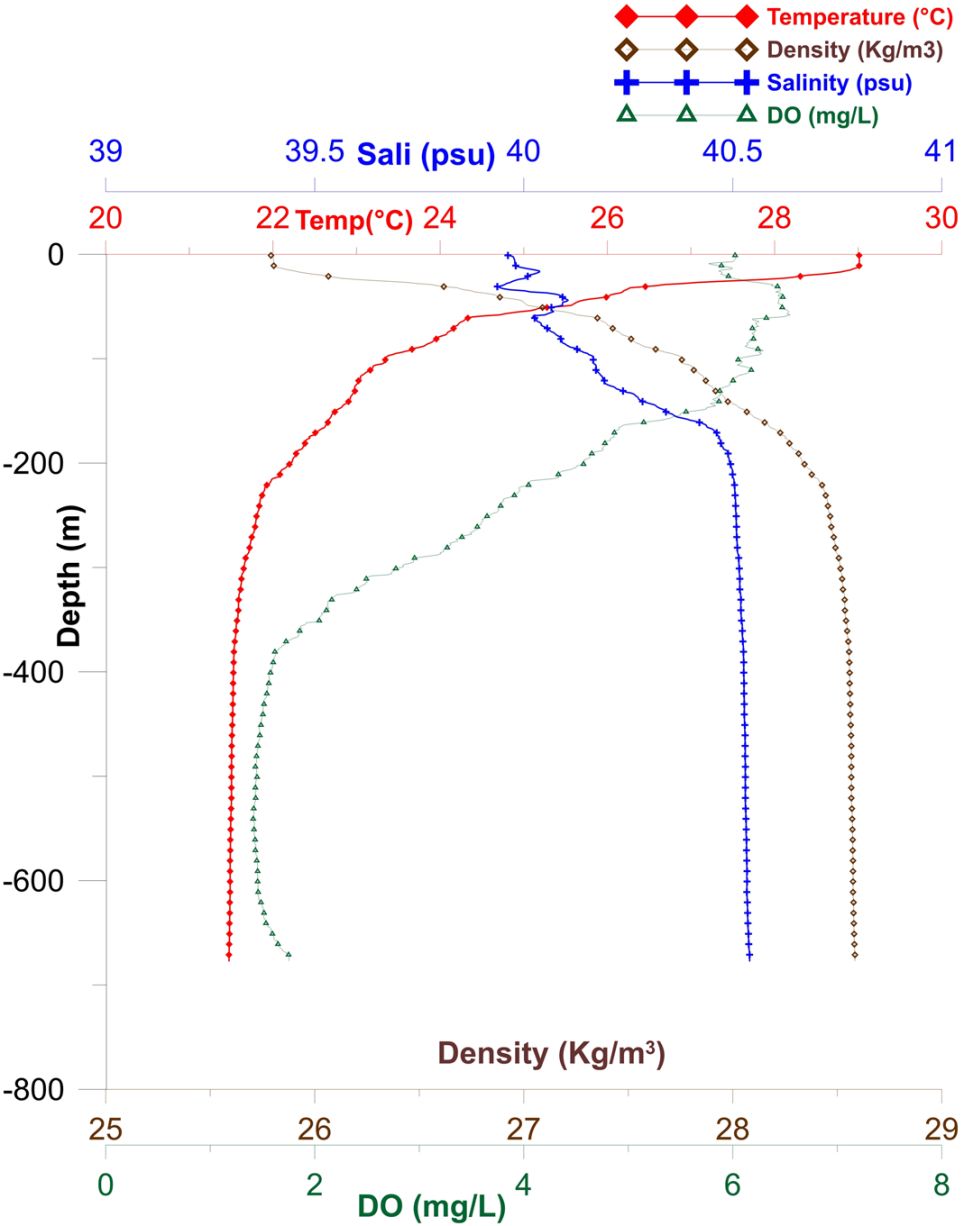
Vertical profile of temperature, salinity, DO, and density at the location where the *E. fistula* framework was found.

The hydrodynamical and hydrographical conditions of the location (Fig. 1) where coral framework was recorded were also studied (Table 1). Relatively strong currents were detected along the entire water column, with a significant decreasing trend from surface (average 36.9 cm/s at 20 m depth) to the bottom (average 8.7 cm s^−1^ at 500 m depth). The surface currents were flowing towards the northwest direction (up to 100 m), while the bottom currents were in the west-southwest direction (Fig. S1). Under experimental conditions for DWC such as *L. pertusa* (now *Desmophyllum pertusum*^31^), low flow speed currents (< 7 cm s^−1^) were found optimal for successful prey capture^32^. Three layers of water masses—a surficial narrow mixed layer (down to 23 m with an average temperature of 29.01 °C), a sharp thermocline (from 23 m down to 200 m with a temperature gradient of 7 °C), and a homogenous bottom layer (from 200 m down to the bottom with a uniform temperature of ~ 21.5 °C) were detected (Fig. 4). Salinity profiles show two high-saline and one low-saline tongues in the subsurface waters between a depth of 20 and 70 m. Dissolved oxygen (DO) content at the surface (6.02 mg L^−1^) increased to 6.5 mg L^−1^ at 50 m. This subsurface maximum DO layer extends down to 150 m, followed by a sharp decreasing trend towards the mid layer with a DO of 1.5 mg L^−1^ at 400 m. A minor increasing trend in the DO level was detected from about 600 m to the seabed (Fig. 4). Yum et al.^21^, reported very low dissolved oxygen content (0.53 mg L^−1^) at a depth of 359 m in the central Red Sea from where *E. fistula* samples were collected and speculated that these corals use mitochondrial hypometabolism and anaerobic glycolysis to manage low oxygen conditions.

Concentrations of all nutrients, except silicon, were in traces within the euphotic zone, while the concentration of nitrate, phosphate, and silicon rapidly increased below the euphotic zone (Table 1). Concentration of Chlorophyll *a* ranged between 0.15 and 0.2 µg L^−1^ within the euphotic zone and increased only at a depth of 65 m, and the total suspended solids (TSS) was around 10 mg L^−1^ irrespective of the depth (Table 1). Chlorophyll maxima (DCM) occur in the deeper layers (> 70 m) with a significantly higher concentration of Chl a than that in the euphotic zone in the area north of 22–24°N^33^, where DWCs were recorded in this study.

The DWCs typically feed on common food items encountered in the deep water habitats, such as zooplankton, phytoplankton, and particulate organic carbon (POC)^34,35^. Sinking surface water in the northern Red Sea flows back to the south as deep-water currents, which may act as a food supplying mechanism for these deep-water corals. Zonal eddies have been reported in the central and northern Red Sea, and these eddies may play a significant role in the injection of nutrients into the water column. The presence of an anticylconic eddy centered around 25°N, with an east-flowing current to the north of 26°N (area of the present study) and a west-flowing current to the south has been reported^36^. The growth of a higher level of phytoplankton in the peripheries of anticyclonic eddies may propagate zooplankton biomass, resulting in high rates of marine snow fall from the periphery of anticyclonic eddies to the DWC framework. Cyanobacterial cells are dominant in the top layers (to a depth of about 20–30 m) of the northern Red Sea, whereas nanoplankton and picoeukaryotes are dominant at depths greater than 30 m and down to the depth of the DCM framework^33^. A study of the isotopic signatures of *E. fistula* collected from the Red Sea by Roder et al.^5^ indicates that *E. fistula* does not exclusively feed on particulate organic matter (POM), and other food sources such as microbes may contribute to the diet of the corals. Qurban et al.^37^ studied the primary production in the northern Red Sea and have speculated that the microbial loop plays a greater role in the trophic dynamics of the Red Sea. Hence, the combined effect of favorable current flow, copious supply of food, and moderate nutrient levels can provide the conditions necessary for the survival and growth of some DWCs in the highly demanding habitat of the deep Red Sea. These DWCs thrive in the deep-sea environment by maximizing the available resources and minimizing the metabolic demands^5^.

Apart from climate change, anthropogenic activities such as bottom trawling, hydrocarbon exploration and/or production, deep sea mining, cable and pipeline installation, pollution, waste disposal, coral exploitation and trade, and destructive scientific sampling threaten the global survival and distribution of DWCs^22,38^. Shipping, marine pollution, oil exploration, offshore infrastructure development for tourism, and aquaculture using floating cages are some of the activities with the potential to negatively impact the DWCs in the Red Sea. Thus, the highest priority regarding these sensitive ecosystems is to locate, map, and protect them by creating marine protected areas (MPAs), similar to the first MPA established off Florida in 1984 for the protection of a cold-water coral habitat^38^. Internationally, DWCs come under the purview of the Vulnerable Marine Ecosystems (VMEs) listed by the United Nations in resolution 61/105 that called upon States to protect VMEs. They also meet the criteria for Ecologically and Biologically Significant Marine Areas (EBSAs) laid down by the Conference of the Parties to the Convention on Biological Diversity (CBD)^38^. Even though recent surveys and studies^5,6,20,21^ have given a fresh impetus to the conservation of deep water corals in the Red Sea, the findings of these studies are yet to receive the attention of local government agencies, conservation organisations, and policy makers. Hopefully, the discovery of framework forming DWCs in the Red Sea and the previously conducted studies on DWCs from 2013^5,6,20,21^ will receive the due recognition of the local policy makers, resulting in well-defined action plans for the conservation and protection of DWC habitats in the Red Sea.

## Methods

An area (26.3843°–26.4335° N; 36.0358°–36.0888° E) located between Al Wajh and Duba in the Saudi waters of the Red Sea (Fig. 1) was randomly chosen for the deep-sea survey. The study area is approximately 30 km from the coast with a depth ranging from 636 to 959 m. A video survey was conducted within a 3 km × 3 km area.

### Video survey and analysis

The survey was conducted on June 30, 2014 using the Research Vessel *R. V. Aegaeo*, belonging to the Hellenic Centre for Marine Research (HCMR). The ROV *Max Rover* (DSSI, USA) available onboard was well equipped for conducting sea bottom surveys and sampling^6^, and it was used for the video survey according to the methods described in Qurban et al^6^. A total of around 12 h of video was recorded during the day of the survey, along the track provided in Fig. 1. Coral samples were collected using the ROV, preserved and brought to the laboratory for further analyses.

The video footage was visually analyzed in the lab to assess the approximate area of coverage of the coral framework. The position of the coral framework was used to develop an ArcGIS distribution map. The coral species was identified based on the specimens collected with the help of taxonomists^13,14^ and DNA analysis.

### DNA analysis

The DNA was extracted from the DWC samples using the Qiagen DNeasy Plant Mini Kit (Qiagen, Hilden, Germany) according to the instructions of the manufacturer. More than 5 replications of the process from extraction to amplification were conducted to ensure that the final sequencing accuracy is more than 99%. The QIAGEN Multiplex PCR Kit (Qiagen, Hilden, Germany) was used to perform the polymerized chain reaction for the amplification of mitochondrial region 16S^39^ and IGR^40^. The PCR cycling parameters used are as follows: initial 95 °C denaturation for 15 min. followed by 35 cycles of 94 °C for 60 s., annealing for 60 s. (LP16SF/LP16SR27: 50 °C; AGAL / DENF: 51 °C), and 72 °C for 60 s., and a final elongation step of 72 °C for 10 min.

The PCR products were checked under UV light after running in 1% agarose gel under 90 V for 45 min. All PCR products were cleaned by incubating with exonuclease I and FastAP Thermosensitive Alkaline Phosphatase (ExoFAP; USB, Cleveland, OH, USA) at 37 °C for 60 min. followed by 85 °C for 15 min. The final products were sequenced in both forward and reverse directions with fluorescently labeled dye terminators according to the manufacturer’s protocols (BigDye, Applied Biosystems Inc., Foster City, CA, USA), and analyzed using an ABI 3130XL Genetic Analyzer (Applied Biosystems). The 16S and IGR sequences were assembled and aligned using the program Geneious R8 (Biomatters Ltd., Auckland, New Zealand) and were uploaded to the GenBank.

### Environmental data collection

A hull-mounted Acoustic Doppler Current Profiler (ADCP, 75 kHz Ocean Surveyor) with deep-water profiling capabilities was used to measure the water current and direction at different depths in and around the DWC area. The ADCP was programmed to profile the entire water column from 20 m to near-bottom at an ensemble interval of 5 min with a bin size of 20 m along the track of the ship. The depth range of good velocity data typically extended to 600 m below the vessel, depending on the conditions of the sea. The ADCP profiling was conducted over an area of 5 km × 5 km in the vicinity of the DWC site.

Vertical profiles of temperature, salinity (conductivity), dissolved oxygen (DO), density, and depth, measured at 1 m intervals, were obtained using a Sea-Bird-9 plus CTD system at nine stations (Fig. 1). Water samples obtained during the CTD cast were used for the determination of nutrients. Concentrations of nitrate, nitrite, phosphate, and silicon were photometrically determined using a SKALAR San Plus model Auto-Analyzer using the analytical methods provided by the manufacturer.

## Supplementary information

Supplementary file1
