# Supplementary material for: Discovery of deep-water coral frameworks in the northern Red Sea waters of Saudi Arabia
Source: Sci Rep. 2020 Sep 18;10:15356. doi: 10.1038/s41598-020-72344-5 (PMC7501261; doi:10.1038/s41598-020-72344-5)
Supplement: Supplementary file 1 — Supplementary file1 [file 41598_2020_72344_MOESM1_ESM.docx]

**Discovery of deep-water coral frameworks in the northern Red Sea waters of Saudi Arabia**

Mohammad A. Qurban^ab^, Periyadan K. Krishnakumar^a*^, Thadickal V. Joydas^a^, Karuppasamy P. Manikandan^a^, T.T.M. Ashraf^a^, Goutham Sambath^a^, Thiyagarajan Duraisamy^a^, Song He^c^, Stephen D. Cairns^d^

^a^ Center for Environment and Water, Research Institute, King Fahd University of Petroleum and Minerals (KFUPM), P. B. No. 391, Dhahran 31261, Saudi Arabia.

^b^ National Center for Wildlife, Ministry of Environment, Water and Agriculture, Saudi Arabia

^c^Red Sea Research Center, Division of Biological and Environmental Science and Engineering, King Abdullah University of Science and Technology, Kingdom of Saudi Arabia

^d^Department of Invertebrate Zoology, National Museum of Natural History, Smithsonian Institution, Washington, DC 20560, United States.

^*^Corresponding author. Tel: +96638607657; fax: +96638601205

Email address: [kkumarpk@kfupm.edu.sa](mailto:kkumarpk@kfupm.edu.sa)

**Additional information -** *Supplementary information*:


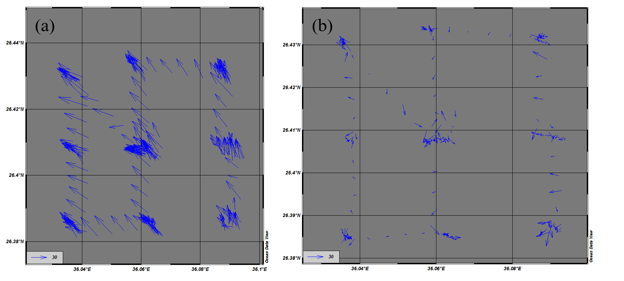


Fig S1. Current vector plot (a) at 20 m depth layer and (b) at 500 m depth layer

**DNA Analysis Results:**

**16s mitochondrial region:**

LP16sF 5’-TTGACCGGTATGAATGGTGT-3’

LP16sR 5’-TCCCCAGGGTAACTTTTATC-3’

From reference Le Goff‐Vitry et al. (2004)

16s Sequence for Deep Sea Coral Samples:

TTTATCTGCTTATCGTTAACTATTTCACCCTAAATTAACGGGTCACTTAAACCCACCTAAAAAGTAAGTGCCTGCTCAGGATTCCCCCTTGGCAGTCAGACATAACTAAAAATATATCTTAAGCCCGCCTTCGTTACTTTTTTAAAGGCGGTCGCCCCAACCAAACTGTCCCACTTATCAAACCCCAAAGACATAACATTTTGAGTTGGCTTTCTTAAAATAAAAGAGTGAGCTTTTCTAACCCGAGAGGTTAACACCACATTTAAAACCTATTTATTTGTAAGAAATAAATTAATTTAAGCCACATAAGTTTCCAGTAAAGTTCCATGGGGACTTCTTGTCTAACAATTTGGATGTAGCATCTTCACTACAAATTCAATTTCACTGGAAATTTCCTTAAGACAGTGAGACCCTCGTGACACCATTCATACCCGGTCAAA

**Mitochondrial intergenic spacer IGR region:**

AGAL 5’ - CGCATTGAAACACGAGCTTA – 3’

DENF 5’ - TTTGCTGGTTGGAATTTGGT – 3’

From reference Arrigoni et al. (2014)

IGR Sequence for Deep Sea Coral Samples:

GAGCTTAAAGCTTCTTCTTCGCTCTTTCGAATCTCCCGTCCAATTTAGCCTATCTTAGTTTTCCCTTTTTCCCCCTTTCCTTTTACCCAAAACTGTAGAGGCGGGAATCGAACCCACTTCTTCGGGGCATGAGCCCGATGACTTACCATTCGTCCTCTCTACAACTTCTTCGAAGTATGAGCTCGAAGAAGCCGGACCCCCCCTCCTAATCAAATCATAAAACTATCAATTAACAAACAAGCGTCCAACACAGTTAAAAACCAAATGAGATGTTTACGCATTCTGTTATACTTCATTCTCTTTGATAAAAGGTAATTCCTCATATGTATGAACTAAAGGAGGAGAAACATGAACCCATTCTAAAGAAGACCAACTCTCCCCGCCTTCATCCGTTCAAGCAACAAATTCCTCTTCTCAAACATATATATCATAAAGAATATATATGAAAAAAATGACTCCTATTATTGAAATAGTAGAACCCAAAGAACTAACCAAATT

Amplified 16S fragments were blasted on GenBank with 99.74% identical match to *Balanophyllia* sp. (HQ439713) from Australia. Our deep-sea coral specimen is a framework forming coral while the genus *Balanophyllia* only contains solitary species (Wood 1844). This means that our specimen cannot be identified as *Balanophyllia* in any cases. For our IGR sequences, the highest match is to *Heteropsammia cochlea* (97.59%) which cannot be our specimen due to the major morphology differences (Hoeksema and Cairns 2019).

There is a 16S sequence of *Eguchipsammia fistula* deposited in Genbank (JX629250), which has been collected from the deep Red Sea (Roder et al. 2013). Our 16S sequences give a 96.51% identical match to this sequence, and the morphological identification agreed with this result. Hence, our sample belongs to *Eguchipsammia fistula*. Since there is no IGR sequence from *Eguchipsammia fistula* in GenBank could be compared with. Our IGR sequence is the first record on this species. Sequences have been deposited in NCBI GenBank under accession numbers [Submission ID: 2328258 16S and 2328249 IGR] (*Eguchipsammia fistula*).

**Reference**

Arrigoni, R. et al. A phylogeny reconstruction of the Dendrophylliidae (Cnidaria, Scleractinia) based on molecular and micromorphological criteria, and its ecological implications. *Zool Scr* **43**, 661-688 (2014).

Hoeksema, B. & Cairns, S. World list of Scleractinia. Scleractinia Accessed through: World Register of Marine Species.Preprint at http://www.marinespecies.org/aphia.php (2019).

Le Goff-Vitry, M., Pybus, O. & Rogers, A. Genetic structure of the deep‐sea coral Lophelia pertusa in the northeast Atlantic revealed by microsatellites and internal transcribed spacer sequences. *Mol. Ecol.* **13**, 537-549 (2004).

Roder, C. et al. First biological measurements of deep-sea corals from the Red Sea. *Sci. Rep.* **3,** 2802 (2013).

Wood, S.V. III.—Descriptive Catalogue of the Zoophytes from the Crag. *J. Nat. Hist.* **13**, 10-21 (1844).
